# Tbet or Continued RORγt Expression Is Not Required for Th17-Associated Immunopathology

**DOI:** 10.4049/jimmunol.1600137

**Published:** 2016-05-11

**Authors:** Verena Brucklacher-Waldert, Cristina Ferreira, Silvia Innocentin, Shraddha Kamdar, David R. Withers, Marika C. Kullberg, Marc Veldhoen

**Affiliations:** * Laboratory of Lymphocyte Signalling and Development, Babraham Institute, Cambridge CB22 3AT, United Kingdom;; † Centre for Immunology and Infection, Department of Biology and Hull York Medical School, University of York, York YO10 5DD, United Kingdom; and; ‡ Medical Research Council Centre for Immune Regulation, University of Birmingham, Birmingham B15 2TT, United Kingdom

## Abstract

The discovery of Th17 cell plasticity, in which CD4^+^ IL-17–producing Th17 cells give rise to IL-17/IFN-γ double-producing cells and Th1-like IFNγ^+^ ex-Th17 lymphocytes, has raised questions regarding which of these cell types contribute to immunopathology during inflammatory diseases. In this study, we show using *Helicobacter hepaticus*-induced intestinal inflammation that IL-17A^Cre^– or Rag1^Cre^-mediated deletion of *Tbx21* has no effect on the generation of IL-17/IFN-γ double-producing cells, but leads to a marked absence of Th1-like IFNγ^+^ ex-Th17 cells. Despite the lack of Th1-like ex-Th17 cells, the degree of *H. hepaticus*-triggered intestinal inflammation in mice in which *Tbx21* was excised in IL-17–producing or Rag1-expressing cells is indistinguishable from that observed in control mice. In stark contrast, using experimental autoimmune encephalomyelitis, we show that IL-17A^Cre^–mediated deletion of *Tbx21* prevents the conversion of Th17 cells to IL-17A/IFN-γ double-producing cells as well as Th1-like IFN-γ^+^ ex-Th17 cells. However, IL-17A^Cre^–mediated deletion of *Tbx21* has only limited effects on disease course in this model and is not compensated by Ag-specific Th1 cells. IL-17A^Cre^–mediated deletion of *Rorc* reveals that RORγt is essential for the maintenance of the Th17 cell lineage, but not immunopathology during experimental autoimmune encephalomyelitis. These results show that neither the single Th17 subset, nor its progeny, is solely responsible for immunopathology or autoimmunity.

## Introduction

The immune system needs to rapidly and robustly respond to pathogenic threats, whereas inappropriate responses to benign stimuli must be avoided. For a long time, the CD4-expressing Th cells that orchestrate adaptive immune responses were thought to consist of two subsets, the Th type 1 (Th1) and Th type 2 (Th2) cells (1). Regulatory T cells (Treg) were identified based on their ability to prevent autoimmunity (2) and were able to reduce the activity of both Th1 and Th2 subsets, thereby upholding the paradigm of two ultimate effector lineage fates. However, in recent years, this paradigm has undergone substantial revision. Upon activation, Ag-inexperienced CD4^+^ T cells can differentiate into multiple lineages, including Th1, Th2, Treg, Th17, Th9, and follicular Th cells (Tfh) (3). The development of these Th subsets is determined by the local environment, and especially, but not exclusively, the cytokines present (4, 5).

Th subsets are largely defined by the signature cytokines they produce and their lineage-associated transcription factors. Thus, Th1 cells are characterized by their expression of the cytokine IFN-γ and the transcription factor T box expressed in T cells (Tbet) (6). Th2 cells express IL-4, -5, -13, and GATA3 (7). Treg cells are defined by the expression of forkhead box p3 (Foxp3) (8), and Th17 cells express IL-17, IL-17F, and RORγt and RORα (9). Each Th subset is often ascribed a specific role in immunity, such as providing help to clear intracellular pathogens (Th1), helminths (Th2), and extracellular bacteria and fungi (Th17) (3). Furthermore, Th subsets also play a prominent role in aberrant immunity. Although Th1 cells were initially thought to be critical in autoimmune disorders such as rheumatoid arthritis, type 1 diabetes, and multiple sclerosis, the focus rapidly shifted to Th17 cells being involved in these diseases (10, 11).

Shortly after the first description of Th17 cells, CD4^+^ T cells producing both IL-17 and IFN-γ (Th1/Th17 or IL-17/IFN-γ double producers) were discovered in both humans and mice (12, 13), their frequency sometimes outnumbering IL-17 or IFN-γ single producers (14). These IL-17/IFN-γ double-producing cells coexpress RORγt and Tbet (15–17). Detailed studies in mice revealed not only the presence of IL-17/IFN-γ double producers (16, 18, 19), but the existence of IFNγ^+^ ex-Th17 cells. Using a fate reporter system in which IL-17–secreting cells are permanently marked, a near complete conversion of Th17 cells to an IFN-secreting Th1-like phenotype could be observed (20). These Th1-like IFNγ^+^ ex-Th17 cells have ceased to express most characteristic factors associated with the Th17 lineage, such as IL-17 and RORγt (16, 19–21), and instead express Tbet and Runt-related transcription factor (Runx) family members (22). The pathogenic potential of Tbet-expressing ex-Th17 cells remains controversial. Mouse models of autoimmunity in which Th17 cells have been implicated in disease pathogenesis have been reported by several laboratories to be dependent on Tbet (23–29), yet others have observed that in vitro polarized Tbet-deficient Th17 cells or Tbet-deficient CD4^+^ T cells maintain a high pathogenic potential (30, 31).

In this study, we investigated whether the Th17 cell lineage and its Tbet- and IFN-γ–expressing progeny are directly responsible for immunopathology during inflammatory responses associated with the Th17 cell lineage. We used two models of inflammation, experimental autoimmune encephalomyelitis (EAE) and the *Helicobacter hepaticus* typhlocolitis model, to examine whether conversion of Th17 cells into Th1-like cells (defined by the expression of Tbet and IFN-γ, and absence of RORγt, IL-17A, and IL-17F) is necessary for immunopathology. The use of an IL-17A-Cre mouse (20) enabled us to track the fate of cells of the Th17 cell lineage as well as conditionally remove genes of interest specifically in IL-17–producing cells and their descendants. As a control, we also made use of a Rag1-Cre mouse to allow us to study the influence of Rag1^Cre^-mediated excision of similar genes. We show that the IL-17A^Cre^– or Rag1^Cre^-mediated removal of *Tbx21* does not impact on the generation of IL-17/IFN-γ double producers, but markedly blocks the generation of Th17 cell–derived Th1-like cells during *H. hepaticus*-induced colitis without reducing immunopathology. During EAE both IL-17/IFN-γ double producers and Th17 cell–derived Th1-like cells are markedly reduced after IL-17A^Cre^–mediated *Tbx21* deletion, but this only modestly reduced immunopathology. Finally, we demonstrate using Rag1^ΔTbet^, Rag1^ΔRORα^, and IL-17A^ΔRORγt^ mice that neither Th17 cell conversion toward Th1-like cells, long-term maintenance of Th17 cells, nor Tbet expression in lymphocytes is essential for the induction of EAE. Together, our findings imply that T cell–associated pathogenicity may not be solely attributed to the Tbet- and IFN-γ–expressing progeny of the Th17 cell lineage.

## Materials and Methods

### Mice

C57BL/6J, IL-17A^Cre^ Rosa^stop-tdRFP^ (20), IL-17A^Cre^ Tbx21^fl/fl^ Rosa^stop-tdRFP^, IL-17A^Cre^ eomesodermin (Eomes)^fl/fl^ Rosa^stop-tdRFP^, Rag1^Cre^ Rosa^stop-tdRFP^ (32), Rag1^Cre^ Tbx21^fl/fl^ Rosa^stop-tdRFP^, Rag1^Cre^ Rorα^fl/fl^ Rosa^stop-tdRFP^, Rag2^−/−^ (33), and IFN-γ^eYFP^ [Yeti; yellow-enhanced transcript for IFN-γ (34)], all on the C57BL6/J strain, were bred at the Babraham Institute. IL-17A^Cre^ Rorγt^fl/fl^ Rosa^stop-tdRFP^ were bred at the University of Birmingham. Tbx21^fl/fl^ and Eomes^fl/fl^ (35) were obtained from S. Reiner (Department of Microbiology and Immunology and Department of Pediatrics, College of Physicians and Surgeons, Columbia University, New York, NY), Rorα^fl/fl^ (36) from A. McKenzie (Medical Research Council Laboratory of Molecular Biology, Cambridge, U.K.), and Rorγt^fl/fl^ from JAX Laboratories. All animals were bred and maintained under specific pathogen-free conditions, and experiments were conducted in accordance with the United Kingdom Scientific Procedures Act (1986) under Project Licenses authorized by the United Kingdom Home Office and local ethical review committees (EAE, Babraham; *H. hepaticus*, York). Animals employed tested negative for Abs to specific murine viruses, including murine norovirus, were free of *Helicobacter spp.* as assessed by PCR, and were >6 wk old when used.

### In vitro T cell cultures

For T cell differentiations, naive CD4^+^CD62L^+^ T cells were isolated from spleens by magnetic beads following the manufacturer’s instructions (Miltenyi Biotec), or by flow cytometric sorting of CD4^+^CD25^−^CD26L^+^CD44^lo^ cells to >98% purity, as previously described (37). Briefly, cells were cultured in IMDM supplemented with 2 mM l-glutamine, 100 U/ml penicillin, 100 μg/ml streptomycin, 5 × 10^−5^ M 2-ME, and 5% FBS. Th17 and Th1 cells were differentiated in 96-well plates coated with 2 μg/ml anti-CD3 (clone 2C11; BioXcell) and 2 μg/ml anti-CD28 (clone 37.51; BioXcell) in the presence of either 20 ng/ml IL-6, 0.2 ng/ml TGF-β1 (PeproTech), 10 μg/ml anti–IFN-γ (clone XMG1.2; BioXcell), and 5 μg/ml anti–IL-4 (clone 11B11; BioXcell) (Th17 condition) or 2 ng/ml IL-12 (PeproTech) and 5 μg/ml anti–IL-4 (Th1 condition). For T cell proliferation, naive CD4^+^ T cells were loaded with 2.5 μM CFSE (Lifesciences).

### EAE induction

For active EAE induction, animals were injected s.c. with 250 μg myelin oligodendrocyte glycoprotein (MOG)_35–55_ peptide (ProImmune) emulsified in IFA (Sigma-Aldrich, Gillingham, U.K.) supplemented with 250 μg *Mycobacterium tuberculosis* extract H37Ra (Difco). The animals also received 200 ng pertussis toxin (List Biological Laboratories) i.p. on days 0 and 2. For passive EAE induction, CD4^+^RFP^+^ cells were sorted by flow cytometry from lymph nodes and spleens of EAE-induced IL-17A^Cre^ Rosa^stop-tdRFP^ mice on day 17 post-MOG peptide immunization, and 2 × 10^5^ CD4^+^RFP^+^ cells (>98% pure) were injected i.v. into Rag2^−/−^ mice. Rag2^−/−^ hosts were injected s.c. with 250 μg MOG_35–55_ peptide (ProImmune) emulsified in IFA (Sigma-Aldrich) supplemented with 250 μg *M. tuberculosis* extract H37Ra (Difco) 5 wk after adoptive transfer. Clinical signs of EAE were assessed blindly and according to the following scores: 0, no signs of disease; 1, flaccid tail; 2, impaired righting reflex and/or gait; 3, partial hind limb paralysis; 4, total hind limb paralysis; and 5, total hind limb paralysis with partial forelimb paralysis.

### *H. hepaticus* infection

To induce typhlocolitis, mice were allocated to treatment groups and inoculated intragastrically with 1.5 × 10^7^ *H. hepaticus* NCI-Frederick isolate 1A (38), isolated from the same mouse colony as isolate *Hh*-1 (American Type Culture Collection strain 51449) (39) and treated i.p. with 1 mg anti–IL-10R (clone 1B1.3a) on days 0 and 7 of *H. hepaticus* infection, as described previously (40). One week after the last mAb injection, mice were sacrificed, and mesenteric lymph nodes (mLN) and large intestines (cecum and colon) were collected for analysis. A piece of ascending colon (∼1 cm from the cecum) was fixed in buffered 10% formalin, and paraffin-embedded sections were stained with H&E (Mary Lyon Centre at MRC Harwell, Oxfordshire, U.K.). Histology sections were evaluated in a blinded fashion using a scoring system based on epithelial hyperplasia and lamina propria (LP) cellularity (0 to 3 each), and goblet cell depletion, submucosal inflammation, edema, crypt abscesses, and ulcers (0 to 1 each). A total score was calculated by adding the individual scores. A typical score for a noninflamed colon is <1.5.

### Cell preparations and flow cytometry

For EAE experiments, single-cell suspensions were prepared from spleens, lymph nodes, lungs, Peyer’s patches, and spinal cord. CNS-infiltrating immune cells were isolated from the spinal cord by isolating the soft tissue from the spine and mashing it through 70-μm mesh filter, followed by 36.5% Percoll (Sigma-Aldrich) separation. For *H. hepaticus* experiments, single-cell suspensions were prepared from mLN. Ceca and colons were cut into 3- to 5-mm pieces and incubated twice in RPMI 1640 containing 10 mM HEPES, 100 U/ml penicillin, 100 μg/ml streptomycin, 2 mM glutamine, 1% FCS, 1 mM DTT, and 5 mM EDTA for 20 min at 37°C while shaking to release epithelial cells. Tissue pieces were then digested with Liberase TL (0.3125 mg/ml; Roche, Burgess Hill, U.K.) and DNase I (125 U/ml; Sigma-Aldrich) in RPMI 1640 containing 10 mM HEPES, 100 U/ml penicillin, 100 μg/ml streptomycin, 2 mM glutamine, and 1% FCS for 1 h at 37°C while shaking. The resulting tissue suspension was passed through a 70-μm cell strainer, centrifuged, resuspended in 40% Percoll, and underlayed with 80% Percoll. After centrifugation at 600 × *g* for 20 min at 10°C, LP cells were recovered from the 40/80% interface and resuspended in medium.

For cytokine profiles, cells were stimulated for 4 h with 500 ng/ml PdBU and 500 ng/ml ionomycin (EAE experiments) or 10 ng/ml PMA and 1 μg/ml ionomcin (*H. hepaticus* experiments) in the presence of brefeldin A (all reagents from Sigma-Aldrich). Cells were stained with anti-CD4, anti-CXCR5, anti-CD44, anti-CD3, and anti-PD1 (EAE experiments) or anti-CD4 and anti-CD3 (*Hh* experiments) and a fixable viability dye, followed by intracellular staining with anti–IL-17A, anti–IL-17F, anti–IFN-γ, anti-TNF, anti–GM-CSF (all BioLegend) (EAE experiments) or anti–IL-17A, anti–IFN-γ, and anti-Tbet (all from eBioscience) (*Hh* experiments). The proportion and absolute numbers of T cells were determined by including counting beads (Spherotech). I-A^b^/MOG_38–49_ tetramer was obtained through the National Institutes of Health Tetramer Facility and used according to their guidelines. Samples were analyzed on a Fortessa 4 flow cytometer (BD Biosciences) (EAE experiments) or a CyAn ADP flow cytometer (Beckman Coulter) (*Hh* experiments), and data were analyzed using FlowJo software (Tree Star).

### Statistical analysis

The *p* values were calculated with a two-tailed Student *t* test. Differences were considered statistically significant with *p* < 0.05. Significance is indicated as follows: *p < 0.05, **p < 0.01, and ***p < 0.001.

## Results

### Immunopathology coincides with appearance of Th17-derived IFN-γ producers

**FIGURE 1.**
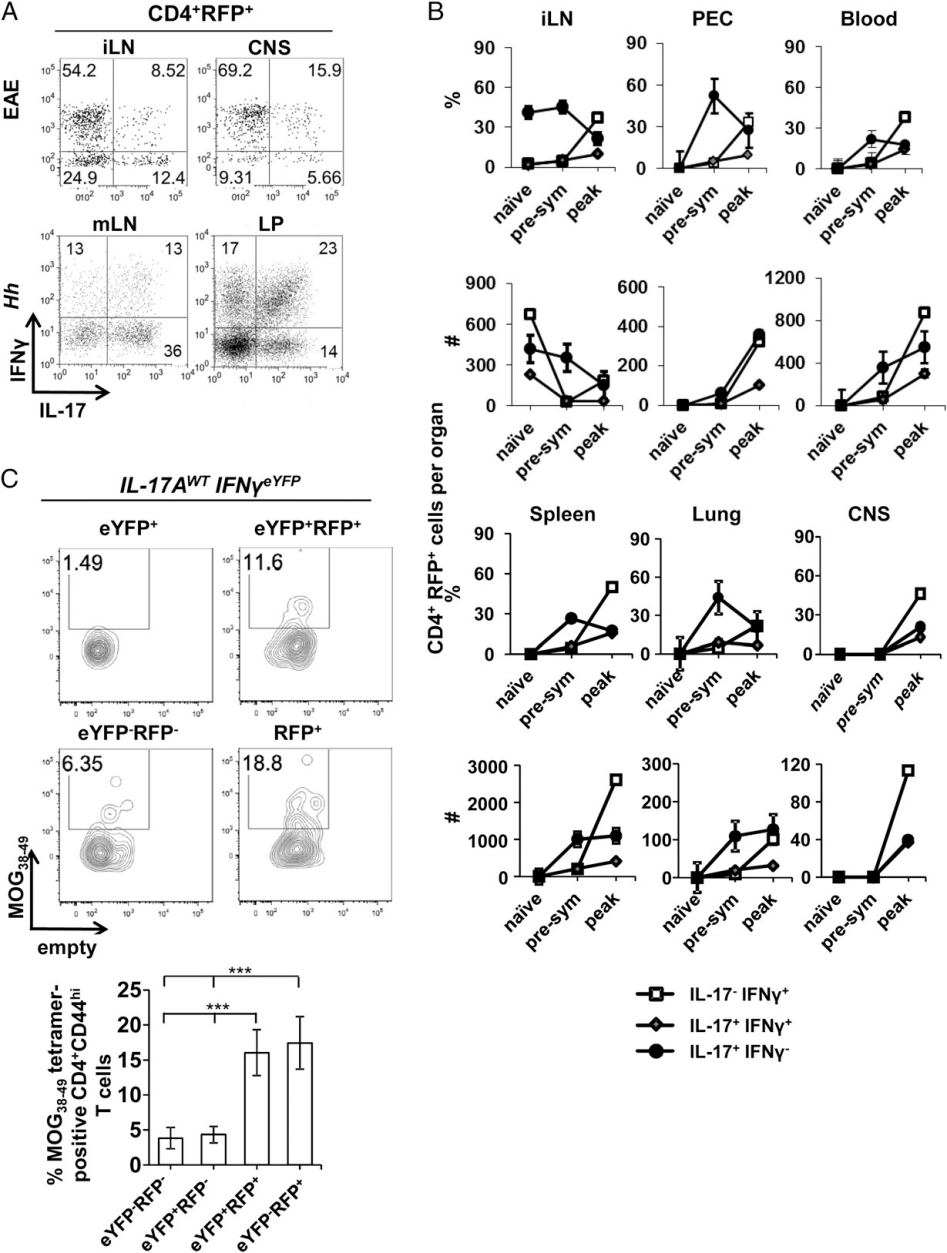
The Th17 cell lineage dominates during inflammation. IL-17A fate-reporter mice (IL-17A^WT^) were subjected to MOG/CFA administration to induce EAE or given *H. hepaticus* (*Hh*) plus anti–IL-10R mAb to induce typhlocolitis, and the cytokine-secreting phenotype of Th17 lineage-positive (RFP^+^) CD4^+^ cells was assessed at different time points. (**A**) Representative intracellular flow cytometry plots for IFN-γ and IL-17 of gated RFP^+^CD4^+^ T cells during EAE (day 17) in iLN and CNS, or during *H. hepaticus* colitis (day 14) in mLN and large intestinal LP. (**B**) Dynamics of Th17 cell–derived populations as a proportion of RFP^+^CD4^+^ T cells (*upper panels*) and their absolute numbers (*lower panels*) in indicated tissues during EAE induction (PEC, peritoneal exudate cells). Naive = prior to MOG/CFA administration, presym = presymptomatic (day 6), and peak = peak of clinical score (day 17). Values represent average ± SEM, *n* = 4/time point. (**C**) Representative staining for I-Ab/MOG_38–49_ (*top flow panels*) and average distribution (*bottom panel*) in indicated T cell populations as proportion of CD4^+^CD44^hi^ T cells harvested from the CNS of IL-17A^WT^IFN-γ^eYFP^ mice at day 17 after EAE induction. Data are representative of two independent experiments (average ± SEM, *n* = 6), ***p < 0.001.

We have previously reported in EAE that Th17-derived Th1-like cells become the dominant T cell population in the CNS (20). To examine the role of Tbet in Th17 cells and their Th1-like progeny in pathology, we made use of two models in which Th17 to Th1-like cell conversion has been established: EAE and the *H. hepaticus* typhlocolitis model (16, 20). Using IL-17A^cre^ Rosa26^stop-tdRFP^ lineage-reporter mice (from hereon called IL-17A^WT^ mice) in which a cell that has turned on IL-17 production is specifically and permanently marked with RFP, we readily detected within the RFP^+^CD4^+^ T cell population the presence of single IL-17 (6–36%), double IL-17/IFN-γ (9–23%), and single IFN-γ producers (13–70%). These cell populations were found in IL-17A^WT^ mice with EAE or *H. hepaticus*-induced colitis both at the site of inflammation (CNS and LP, respectively) and in draining lymph nodes (Fig. 1A). Under steady state conditions in the large intestine, the proportions of IL-17– or IFN-γ–producing CD4^+^ T cells average at 3–4%, indicating that the enhanced frequencies of cytokine-positive CD4^+^ T cells observed in the intestine are induced following *H. hepaticus* inoculation (data not shown) (16). To gain insight into the generation and distribution of these three subpopulations, we performed a kinetic analysis upon EAE induction. We analyzed IL-17 and IFN-γ expression in RFP^+^ CD4^+^ T cells of IL-17A^WT^ mice in draining (inguinal) lymph nodes, the peritoneal cavity, blood, spleen, lungs, and the CNS before MOG_35–55_ immunization, at day 6 prior to onset of clinical signs (presym), and at day 17 during established EAE (peak). In secondary lymphoid tissues, peritoneal cavity, blood, and lung, the fraction of Th17 cells (IL-17 single positive) within the RFP^+^CD4^+^ T cell compartment increased during the presymptomatic phase, and regressed at the peak of the disease in favor of IL-17/IFN-γ double-producing cells and IFN-γ–producing ex-Th17 cells (Fig. 1B). In the CNS, all three cell populations were detectable at their highest levels at the peak of the disease (Fig. 1B). We confirmed that RFP^+^ cells were the majority of CD4^+^ T cells present in the CNS at the peak of the disease, and 46.1% ± 17.41 of these RFP^+^ cells were IFN-γ single-producing ex-Th17 cells (data not shown). The total number of Th17 cells in inguinal lymph nodes (iLN) declined during peak of clinical EAE symptoms, whereas in all other organs total Th17 cell numbers were highest at the peak of the disease (Fig. 1B). IL-17/IFN-γ double-producing T cell numbers remained low before disease onset, and increased during the peak clinical phase in all organs (Fig. 1B). Numbers of ex-Th17 cells, with a Th1-like profile (RFP^+^, IFN-γ^+^, IL-17^−^), increased during peak clinical scores in all organs (Fig. 1B) and also outnumbered bona fide Th1 cells in the iLNs, blood, spleen, and CNS (data not shown) (20). Thus, the majority of CD4^+^ T cells present in the target organ during EAE in IL-17A^WT^ mice are Th17 cells, and their progeny have converted to an IL-17/IFN-γ double-producing or Th1 cell-like phenotype. The appearance of these latter two populations coincides with the onset and maintenance of clinical disease.

To determine what cell population harbors Ag-specific T cells, we used MOG_38–49_ MHC-II tetramer staining in IL-17A^cre^ Rosa26^stop-tdRFP^ IFN-γ^eYFP^ mice (from hereon called IL-17A^WT^IFN-γ^eYFP^ mice) in which IL-17 is lineage marked by RFP and IFN-γ protein expression reported via eYFP (20, 41). We determined the proportion of MOG_38–49_ MHC-II tetramer-positive cells within activated CD4^+^CD44^hi^ T cells that were negative for RFP and eYFP, single positive for either, or positive for both. In line with an important role for Th17 cells in the initiation of EAE (11), the majority of Ag-specific CD4^+^ T cells in the CNS was found within the Th17 cells (eYFP^−^RFP^+^) and the Th17 cell–derived IL-17/IFN-γ double or IFN-γ single producers (eYFP^+^RFP^+^), but not in bona fide Th1 cells (eYFP^+^RFP^−^) (Fig. 1C).

### For *H*. *hepaticus*–induced intestinal pathology, full conversion of Th17 to Th1 is not required

**FIGURE 2.**
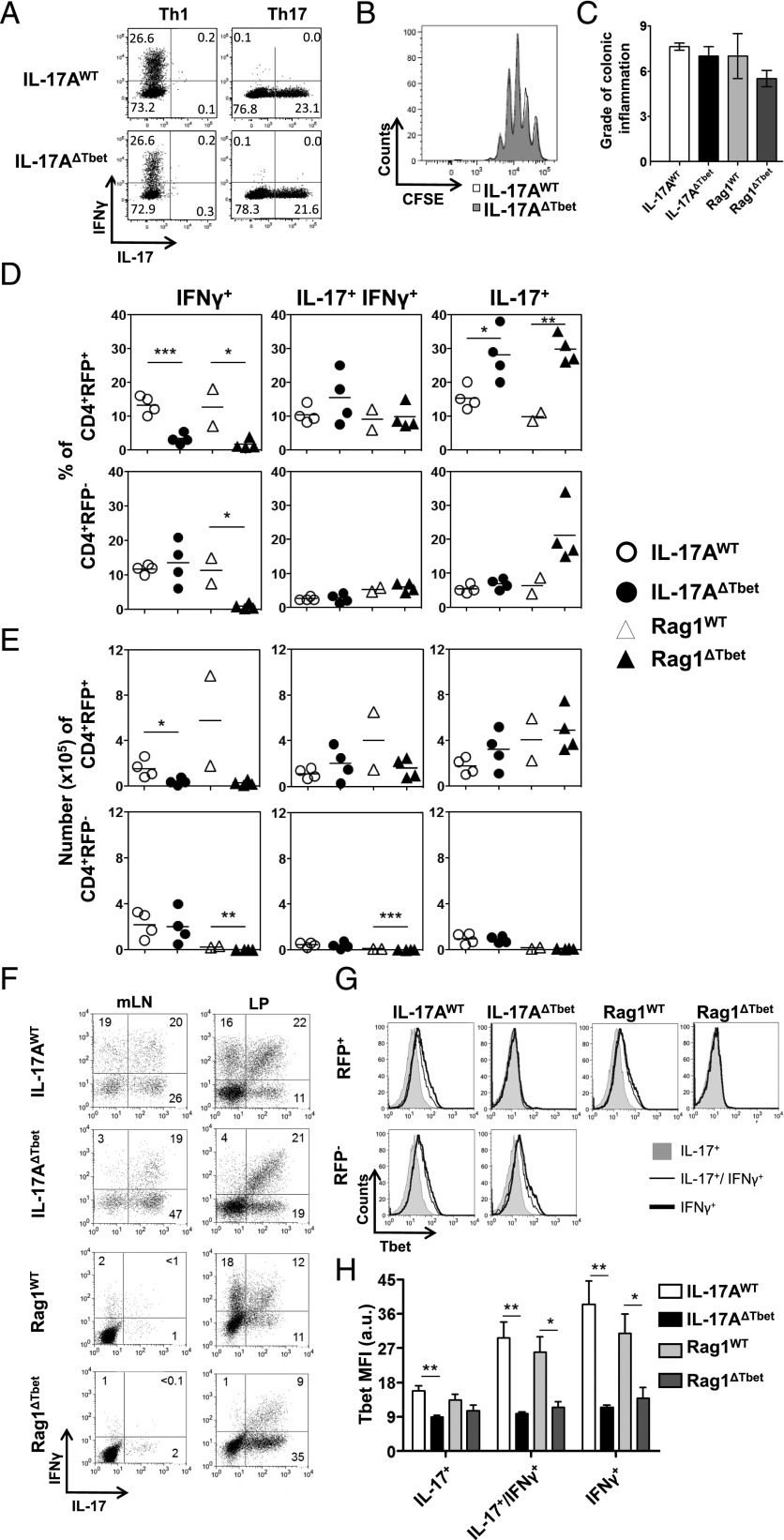
Th17 to Th1 conversion is not required for *H. hepaticus*-induced intestinal pathology. IL-17A^WT^ mice were crossed with floxed *Tbx21* mice and their naive T cell polarization potential assessed in vitro (**A** and **B**), and IL-17A^WT^, IL-17A^ΔTbet^, Rag1^WT^, and Rag1^ΔTbet^ mice were inoculated with *H. hepaticus* (*Hh*) plus anti–IL-10R, and, 2 wk later, ceca, colons, and mLN were collected and processed for histology (**C**) and/or intracellular staining for cytokines and Tbet (**D**–**H**). (A) In vitro differentiation of naive CD4^+^ T cells from IL-17A^WT^ controls and IL-17A^ΔTbet^ mice toward Th1 and Th17 lineages. (B) Proliferation profile of naive CD4^+^ T cells cultured under Th17-polarizing conditions from indicated mouse lines. (C) Histology scores of ascending colon from indicated mouse lines 2 wk post-*Hh*/anti–IL-10R administration (*n* = 4 per group except for Rag1^WT^ where only two mice were examined). Data for IL-17A^WT^ and IL-17A^ΔTbet^ mice are representative of two independent experiments. (D and E) Proportions (D) and numbers (E) of LP RFP^+^CD4^+^ and RFP^−^CD4^+^ T cells expressing IFN-γ alone, IL-17 alone, or both IFN-γ and IL-17 from pooled cecum and colon from indicated mouse lines 2 wk post-*Hh*/anti–IL-10R administration. Data for IL-17A^WT^ and IL-17A^ΔTbet^ mice are representative of two independent experiments. (F) Dot plots show the proportions of IL-17 single-positive, IL-17/IFN-γ double-positive, and IFN-γ single-positive cells within the mLN and LP RFP^+^CD4^+^ T cell population from indicated mouse lines 2 wk post-*Hh*/anti–IL-10R administration. (G) Assessment of Tbet expression by flow cytometry staining in RFP^+^ (*top row*) or RFP^−^ (*bottom row*) LP CD4^+^ T cells positive for either IL-17 alone (filled gray), IL-17 and IFN-γ (thin line), or IFN-γ alone (bold line) from indicated mouse lines 2 wk post-*Hh*/anti–IL-10R administration. (H) Average mean fluorescence intensity (MFI) of Tbet staining of indicated RFP^+^ CD4^+^ T cell subsets derived from indicated mouse lines. Data are from the mice shown in (D) and (E) (averages ± SEM). *p < 0.05, **p < 0.01, ***p < 0.001.

To examine the importance of the Th17 to Th1-like cell conversion for the onset and progression of immunopathology, we generated mice in which *Tbx21* is conditionally deleted upon IL-17 expression and in which IL-17–producing T cells are permanently marked by RFP (IL-17A^Cre^ Tbx21^fl/fl^ Rosa26^stop-tdRFP^, from hereon called IL-17A^ΔTbet^). The IL-17A^ΔTbet^ mice exhibited normal gross development and were born according to a Mendelian distribution (data not shown). Moreover, we confirmed efficient and specific *Tbx21* excision in RFP^+^ cells from IL-17A^ΔTbet^ mice by PCR (data not shown). The in vitro differentiation potential of naive CD4^+^ T cells toward the Th1 or Th17 cell lineages as well as their proliferation were similar in IL-17A^ΔTbet^ and IL-17A^WT^ control mice (Fig. 2A, 2B), demonstrating that polarization toward Th17 and Th1 cells was not affected by the IL-17A^Cre^–mediated removal of *Tbx21*.

Th17-derived Th1-like cells have been detected in *H. hepaticus*-induced intestinal inflammation in which their generation correlates with the development of pathology (16). To examine the role of Tbet in Th17 to Th1 conversion in this model, IL-17A^WT^ and IL-17A^ΔTbet^ mice were given *H. hepaticus* plus anti–IL-10R mAb to induce typhlocolitis, and colonic inflammation was examined 2 wk later. To exclude the potential initiation of Th17 to Th1 cell conversion prior to *Tbx21* excision giving rise to IL-17/IFN-γ double-producing T cells, we also included in these experiments Rag1^Cre^ Rosa^stop-tdRFP^ and Rag1^Cre^ Tbx21^fl/fl^ Rosa^stop-tdRFP^ mice (from hereon called Rag1^WT^ and Rag1^ΔTbet^ mice). Rag1^ΔTbet^ mice allowed us to study the influence of Rag1-mediated *Tbx21* excision on the development of *H. hepaticus*-induced pathology and Th17 conversion. Our findings demonstrate that the degree of colonic pathology was indistinguishable between *H. hepaticus*/anti–IL-10R–treated IL-17A^WT^, IL-17A^ΔTbet^, Rag1^WT^, and Rag1^ΔTbet^ mice (Fig. 2C). Within the CD4^+^ T cells not derived from Th17 cells (RFP^−^ cells), we did not observe any differences in proportion or cell numbers expressing IL-17 or IFN-γ between IL-17A^ΔTbet^ and IL-17A^WT^ controls (Fig. 2D, 2E). As expected, very few RFP^−^CD4^+^ T cells were observed in Rag1^WT^ and Rag1^ΔTbet^ mice (Fig. 2E). When examining the RFP^+^ population, the percentage of LP IL-17 single-producing Th17 cells was significantly enhanced in IL-17A^ΔTbet^ compared with IL-17A^WT^ animals and in Rag1^ΔTbet^ compared with Rag1^WT^ mice (Fig. 2D). However, upon IL-17A^Cre^–mediated *Tbx21* deletion, CD4^+^ T cells in mLN and LP failed to fully switch to Th1-like cells (Fig. 2F), and both the percentage and number of RFP^+^ Th1-like cells were almost absent (90% reduction) in *H. hepaticus*/anti–IL-10R–treated IL-17A^ΔTbet^ compared with IL-17A^WT^ mice (Fig. 2D, 2E). A similar picture was observed in the LP of Rag1^ΔTbet^ mice (Fig. 2D–F). It could be possible for bona fide Th1 cells to compensate for the reduction in Th17-derived Th1-like cells in the IL-17A^ΔTbet^ mice. However, we found no difference in IFN-γ–producing CD4^+^RFP^−^ cell proportions or numbers between this strain and IL-17A^WT^ mice (Fig. 2D, 2E). That *Tbx21* excision had worked efficiently in the two ΔTbet strains was confirmed by flow cytometry showing the absence of Tbet staining in IFN-γ and IL-17/IFN-γ–producing CD4^+^RFP^+^ populations of IL-17A^ΔTbet^ and Rag1^ΔTbet^ mice, but the presence of Tbet in the CD4^+^RFP^+^ cells from IL-17A^WT^ and Rag1^WT^ animals (Fig. 2G, 2H). Together, these data indicate that colonic immunopathology during *H. hepaticus*-induced typhlocolitis does not depend on the generation of Tbet- and IFN-γ–expressing ex-Th17 cells or the activity of Tbet; however, a role for IL-17/IFN-γ double producers cannot be excluded.

### Conditional deletion of Tbet prevents Th17 to Th1 cell conversion in EAE

**FIGURE 3.**
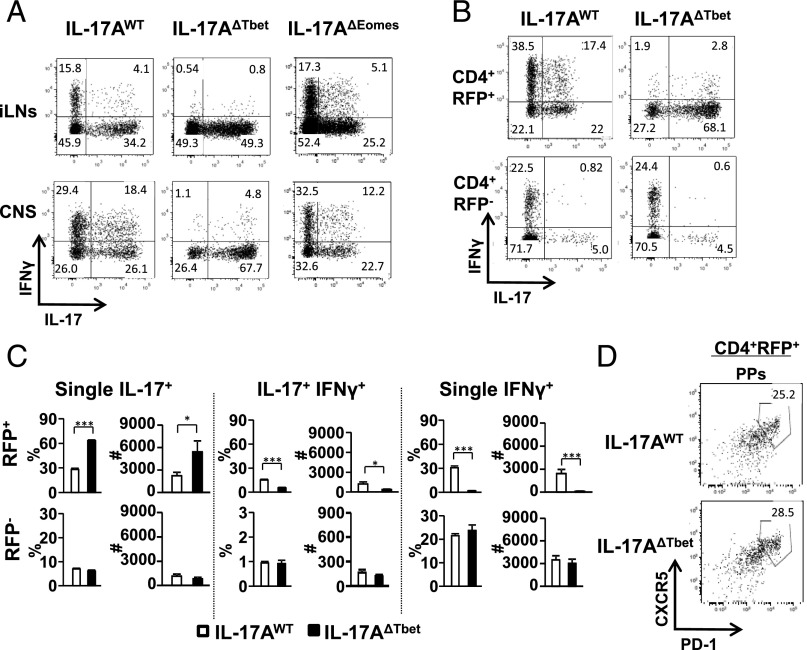
Tbet is required for Th17 to Th1 conversion in EAE. IL-17A fate-reporter mice (IL-17A^WT^) were crossed with floxed *Tbx21* or Eomes mice. T cells were sourced from the iLN or CNS of IL-17A^WT^ controls, IL-17A^ΔTbet^, and IL-17A^ΔEomes^ mice at the onset of EAE symptoms (day 17) and characterized for cytokine production (**A**–**C**), or from the Peyer’s patches of nonchallenged mice (**D**). (A) Flow cytometry for IFN-γ and IL-17 in RFP^+^ Th17 lineage–positive cells in indicated mouse lines and tissues 17 d after EAE induction. (B) Representative dot plots of RFP^+^ (*top row*) or RFP^−^ (*bottom row*) CD4^+^ T cells harvested from the CNS at day 17 post-EAE induction from IL-17A^WT^ controls (*left panels*) and IL-17A^ΔTbet^ mice (*right panels*) and stained for IFN-γ and IL-17. (C) RFP^+^ Th17 lineage-positive (*top panels*) and RFP^−^ lineage-negative (*bottom panels*) cells from IL-17A^WT^ controls (open bars) or IL-17A^ΔTbet^ mice (black bars) were stained for IL-17 and IFN-γ, and proportions (*left panels*) and cell numbers (*right panels*) of cells expressing IL-17 and/or IFN-γ are shown. (D) Staining for PD-1 and CXCR5 in Peyer’s patches of indicated mouse lines. Dot plots are gated on RFP^+^CD4^+^ cells. Data are from two independent experiments with *n* = 4–5 per experiment (averages ± SEM). *p < 0.05, ***p < 0.001.

**FIGURE 4.**
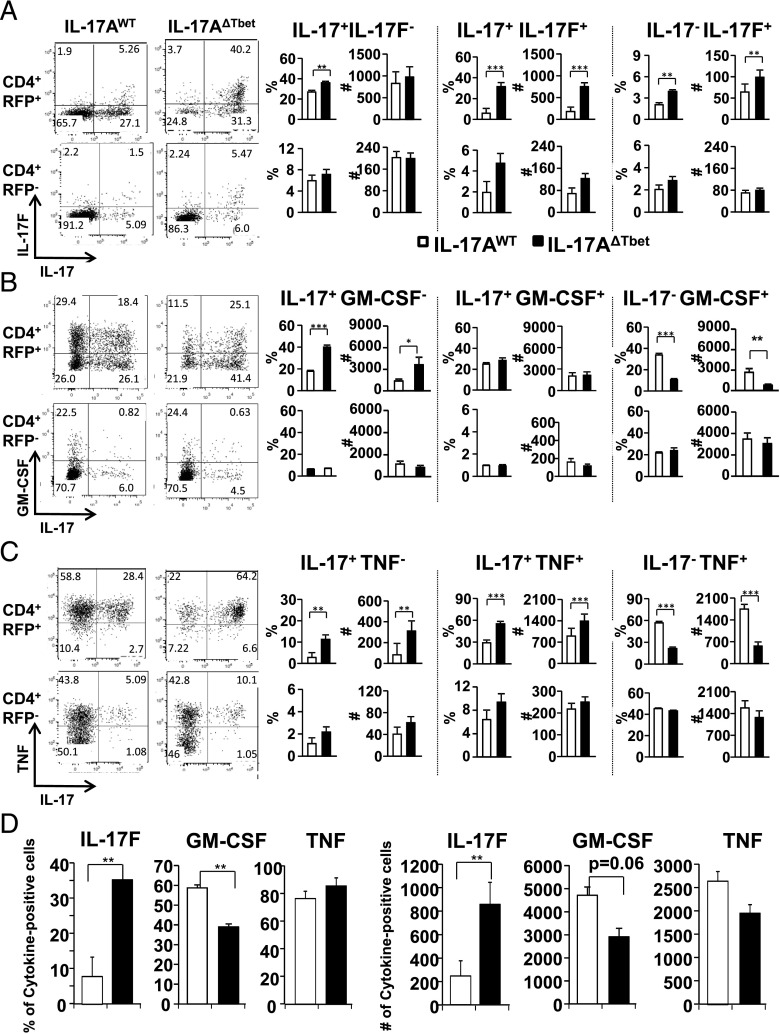
Characterization of Tbet-deficient Th17 cells in EAE. T cells were sourced from the CNS of IL-17A^WT^ controls and IL-17A^ΔTbet^ mice at the peak of EAE symptoms (day 15–17) and characterized for their cytokine production (**A**–**D**). RFP^+^ Th17 lineage-positive (*top panels*) and RFP^−^ lineage-negative (*bottom panels*) cells from IL-17A^WT^ controls (white bars) or IL-17A^ΔTbet^ mice (black bars) were stained for IL-17, GM-CSF, and TNF, and proportions (*left panels*) and cell numbers (*right panels*) of cells expressing IL-17 and/or IL-17F (A), GM-CSF (B), and TNF (C) are shown. (D) Total proportion and numbers of IL-17F–, GM-CSF–, and TNF-producing CD4^+^ T cells present in the CNS of indicated mouse lines (averages ± SEM, *n* = 4–5). *p < 0.05, **p < 0.01, ***p < 0.001.

The role of specific CD4^+^ Th subsets in EAE pathogenesis remains poorly understood with conflicting findings in the literature [for review, see (42)], but so-called polyfunctional T cells or IL-17/IFN-γ double-producing CD4^+^ T cells have been implicated in the disease process (43). To examine the role of Tbet in Th17 cell plasticity in EAE, we performed a detailed analysis of lineage-marked Th17 cells and their progeny in IL-17A^WT^ versus IL-17A^ΔTbet^ mice. Upon MOG_35–55_ immunization, Th17 cells were readily detected in the iLN and CNS of IL-17A^WT^ and IL-17A^ΔTbet^ mice (Fig. 3A). Compared with the IL-17A^WT^ hosts, we found an enhanced proportion and a 3-fold increase in the number of IL-17 (and IL-17F) single-producing cells in the CNS of IL-17A^ΔTbet^ mice 17 d post-MOG peptide administration, indicating a greater stability of the IL-17–producing Th17 cell profile upon *Tbx21* excision (Figs. 3B, 3C, 4D). Consistent with the findings in *H. hepaticus*-induced colitis, Th17-derived IFN-γ^+^ Th1-like cells were absent in IL-17A^ΔTbet^ mice compared with IL-17A^WT^ controls at this same time point after EAE induction (Fig. 3A–C). However, in marked contrast to *H. hepaticus*-induced colitis, both the proportion and number of IL-17/IFN-γ double-producing T cells were reduced (>95%) during EAE (Fig. 3A–C). As Eomes can be an important mediator of T cell IFN-γ expression and T cell cytotoxicity (44), we next used mice in which Eomes is conditionally deleted in IL-17–expressing cells (IL-17A^ΔEomes^ mice). Our data show that the proportions of IL-17– and IFN-γ–producing T cells were indistinguishable in IL-17A^ΔEomes^ and IL-17A^WT^ control mice (Fig. 3A). Together, these results demonstrate that Tbet, but not Eomes, is required for the efficient conversion of Th17 cells to IL-17/IFN-γ double-producing and IFN-γ single-producing CD4^+^ T cells in the EAE model.

As Th17 cells have also been shown to convert to Tfh in the intestine (45), we next analyzed Th17-derived Tfh cells in Peyer’s patches in nonimmunized IL-17A^WT^ versus IL-17A^ΔTbet^ mice. Th17-derived Tfh cells (identified as CD4^+^RFP^+^CXCR5^+^PD-1^+^ cells) were detected in similar proportions in Peyer’s patches of IL-17A^WT^ controls and IL-17A^ΔTbet^ mice (Fig. 3D), together suggesting that Tbet is not required for the conversion of Th17 cells to Tfh cells. Furthermore, it highlights that Tbet is not required for the conversion of Th17 cells per se, but only for the generation of IFN-γ–producing Th1-like cells. These results indicate that the IL-17A^ΔTbet^ mouse is a promising model to specifically study the role of Th17 to Th1 plasticity in autoimmunity and infection.

### Tbet-deficient Th17 cell populations have an altered cytokine profile

Because Th17 cells and IL-17/IFN-γ double-producing T cells have been implicated in the pathogenesis of EAE, we analyzed the cytokine profile of the Th17 cell lineage in the presence or absence of Tbet. Seventeen days after induction of EAE, the CD4^+^ T cell populations present in the CNS were analyzed for their cytokine profile by flow cytometry. As expected, inflammatory cytokines assayed, with the exception of GM-CSF, were enriched within the RFP^+^ Th17 cell lineage compared with the RFP^−^ population (Fig. 4A–C) (20). No significant difference in cytokine production by the RFP^−^ non-Th17 lineage-derived cells was found between IL-17A^WT^ and IL-17A^ΔTbet^ hosts (Fig. 4A–C).

The prevention of Th17 to Th1 cell conversion in IL-17A^ΔTbet^ mice resulted in changes in cytokine profiles of the Th17 cell–derived populations. IL-17F–expressing cells were significantly increased in the Tbet-deficient Th17 cell population (Fig. 4A, 4D). This increase in number was found in all RFP^+^ Th17 cell–derived populations independent of their IL-17 expression profile. The proportion and number of GM-CSF–expressing T cells, a cytokine strongly associated with autoimmunity and required for the induction of EAE (46), were significantly altered in the Th17 cell population from IL-17A^ΔTbet^ mice compared with controls. Thus, in the absence of Tbet, there were more IL-17^+^ GM-CSF^−^ RFP^+^ cells, whereas in the presence of Tbet there were more IL-17^−^ GM-CSF^+^ RFP^+^ cells (Fig. 4B). Although the total proportion of GM-CSF–expressing CD4^+^ T cells was reduced, the total number of cells expressing GM-CSF was not significantly altered when the Th17 cell subset was Tbet sufficient or deficient (Fig. 4D). Expression of TNF followed a similar pattern as GM-CSF. In the absence of Tbet, there were more IL-17^+^ TNF^−^ RFP^+^ cells, whereas in the presence of Tbet there were more that have lost their IL-17 expression (IL-17^−^TNF^+^RFP^+^) cells (Fig. 4C). The combination of RFP^+^ cells expressing both IL-17 and TNF was significantly higher in the IL-17A^ΔTbet^ mice compared with the IL-17A^WT^ controls (Fig. 4C). The overall proportion of TNF-producing cells was, however, not significantly different in CD4^+^ T cells from IL-17A^ΔTbet^ mice (85.4 ± 5) compared with IL-17A^WT^ controls (76.5 ± 6) (Fig. 4D). These results suggest that TNF and GM-CSF expression do not depend on the expression of Tbet and may precede the conversion of Th17 to Th1-like cells. Furthermore, it shows that Tbet expression alters the combination of cytokines simultaneously expressed by the same T cell, but does not affect the total number of TNF- or GM-CSF–producing CD4^+^ T cells present in the CNS.

### Th17 to Th1 cell conversion is not required for EAE pathogenesis

**FIGURE 5.**
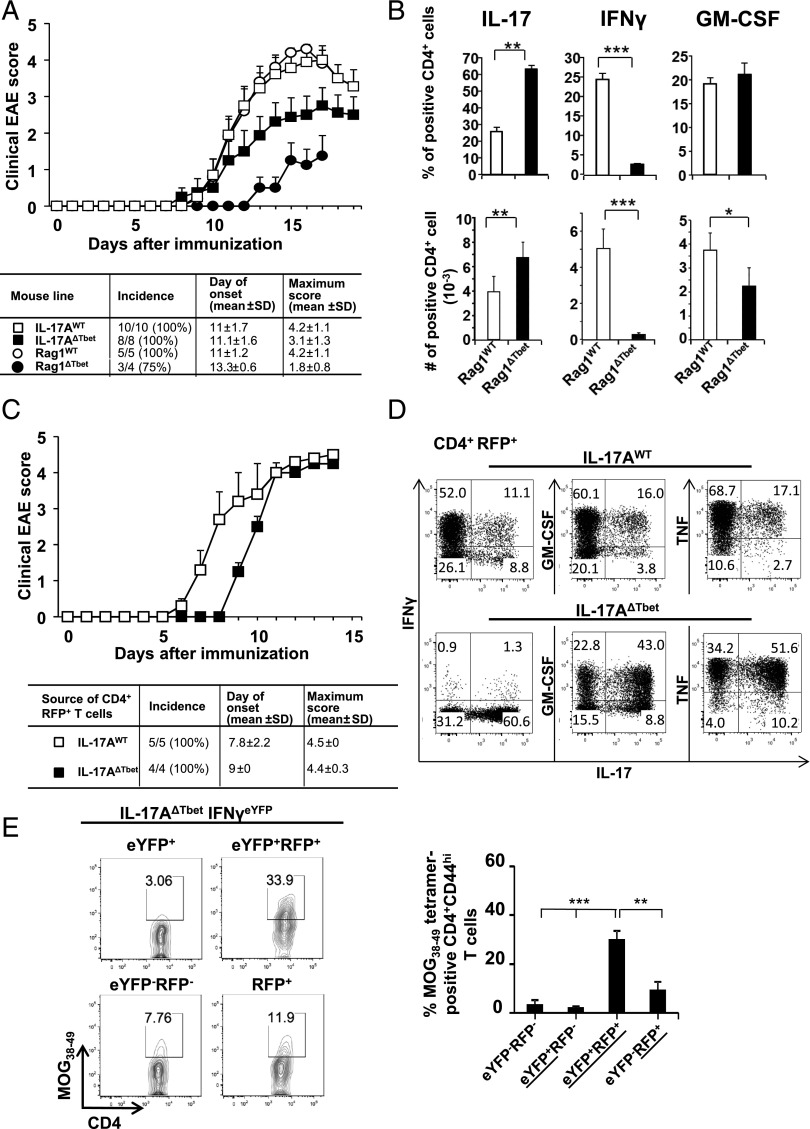
Th17 to Th1 conversion is not required for EAE pathogenesis. (**A**) Clinical EAE scores of four indicated mouse lines immunized with MOG/CFA (*n* = 8–9/group, two biological repeats). (**B**) Relative distribution (*top panels*) and number of cells (*lower panels*) expressing indicated cytokines in the CNS of IL-17A^WT^ controls (white bars) or IL-17A^ΔTbet^ (black bars) during EAE (day 17). (**C**) Clinical EAE scores of Rag2^−/−^ mice, upon adoptive transfer of flow-sorted CD4^+^RFP^+^ T cells obtained from indicated mouse lines, immunized with MOG/CFA (*n* = 6/group pooled from two biological repeats). (**D**) Flow cytometry for indicated cytokines on cells obtained from mice undergoing EAE, as shown in (C). (**E**) Staining for I-Ab/MOG_38–49_ in indicated T cell populations (*left panels*) harvested from the CNS and proportional distribution (*right panel*) as proportion of CD4^+^CD44^hi^ T cells (*n* = 8). Data are pooled from two independent experiments (average ± SEM). *p < 0.05, **p < 0.01, ***p < 0.001.

As IL-17/IFN-γ double-producing T cells have been associated with autoimmune and inflammatory pathology (19, 21, 47, 48), we next investigated the susceptibility of the IL-17A^ΔTbet^ mouse to EAE. We found no difference with respect to timing of EAE onset between IL-17A^ΔTbet^ and IL-17A^WT^ controls (Fig. 5A). However, the maximum clinical scores were reduced in IL-17A^ΔTbet^ hosts (Fig. 5A). As Tbet and other factors implicated in immunopathology, such as GM-CFS, are not exclusively expressed by the Th17 cell lineage, we next assessed the susceptibility of Rag1^ΔTbet^ mice in which Tbet was conditionally deleted via Rag1-Cre in all lymphocytes. In this case, Rag1^ΔTbet^ mice showed a more pronounced reduction in EAE susceptibility, with later onset and lower maximum clinical score than IL-17A^WT^, Rag1^WT^ controls, and IL-17A^ΔTbet^ mice (Fig. 5A). This finding indicates that blocking Th17 to Th1 cell conversion as well as de novo Th1 cell differentiation had a more pronounced impact on reducing EAE pathogenesis than removal of Tbet in IL-17–expressing cells only. However, we cannot exclude an additional role for Tbet in other lymphocytes that once expressed Rag1. As we observed in the IL-17A^ΔTbet^ animals, Rag1^ΔTbet^ mice showed an increased proportion and number of IL-17–producing cells and a marked decrease in the proportion and number of IFN-γ–producing CD4^+^ T cells in the CNS at day 17 post-MOG immunization (Fig. 5B). Although no difference in the proportion of GM-CSF–producing T cells was observed in Rag1^ΔTbet^ mice compared with Rag1^WT^ controls, the number of GM-CSF–producing CD4^+^ T cells in the CNS was reduced in the former animals (Fig. 5B). The reduction in IFN-γ– and GM-CSF–producing T cells in the CNS of Rag1^ΔTbet^ mice correlated with reduced maximum EAE scores (Fig. 5A).

We subsequently investigated whether the block in Th17 to Th1 cell conversion was maintained long-term in vivo. Seventeen days following MOG_35–55_ immunization of IL-17A^WT^ and IL-17A^ΔTbet^ mice, RFP^+^CD4^+^ T cells were isolated from the draining iLNs and transferred to Rag2^−/−^ hosts. Upon subsequent MOG_35–55_/CFA immunization of the recipient mice, a delayed onset of EAE was observed in the group receiving IL-17A^ΔTbet^ RFP^+^CD4^+^ T cells, although equally high clinical scores were observed in both host groups (Fig. 5C). Furthermore, the majority of IL-17A^WT^ control cells had converted to a Th1-like IFN-γ–expressing phenotype (Fig. 5D), whereas IL-17A^ΔTbet^ cells remained stable in their IL-17–expressing profile and did not express IFN-γ (Fig. 5D). The distribution of TNF and GM-CSF was also similar to that seen in the respective donor mice (Fig. 4B, 4C), with the majority of GM-CSF– and TNF-producing cells found among the IFN-γ–producing cells in IL-17A^WT^ controls, but within IL-17–producing cells in IL-17A^ΔTbet^ cells (Fig. 5D).

Although the majority of CD4^+^ T cells encountered in the CNS in both IL-17A^ΔTbet^ and IL-17A^WT^ control mice were originally derived from the Th17 cell subset, as we reported before in IL-17A^WT^ control mice (20), it was possible that Ag specificity could have risen in the bona fide Th1 cell population. However, MOG_38–49_ tetramer staining in IL-17A^ΔTbet^IFNγ^eYFP^ mice at day 17 after EAE induction revealed that Ag specificity remained within the RFP^+^ populations, as previously seen in IL-17A^WT^IFN-γ^eYFP^ control mice (Fig. 1C), but was particularly enriched within the few remaining IL-17/IFN-γ double producers (Fig. 5E).

### RORγt is required to maintain Th17 cells

**FIGURE 6.**
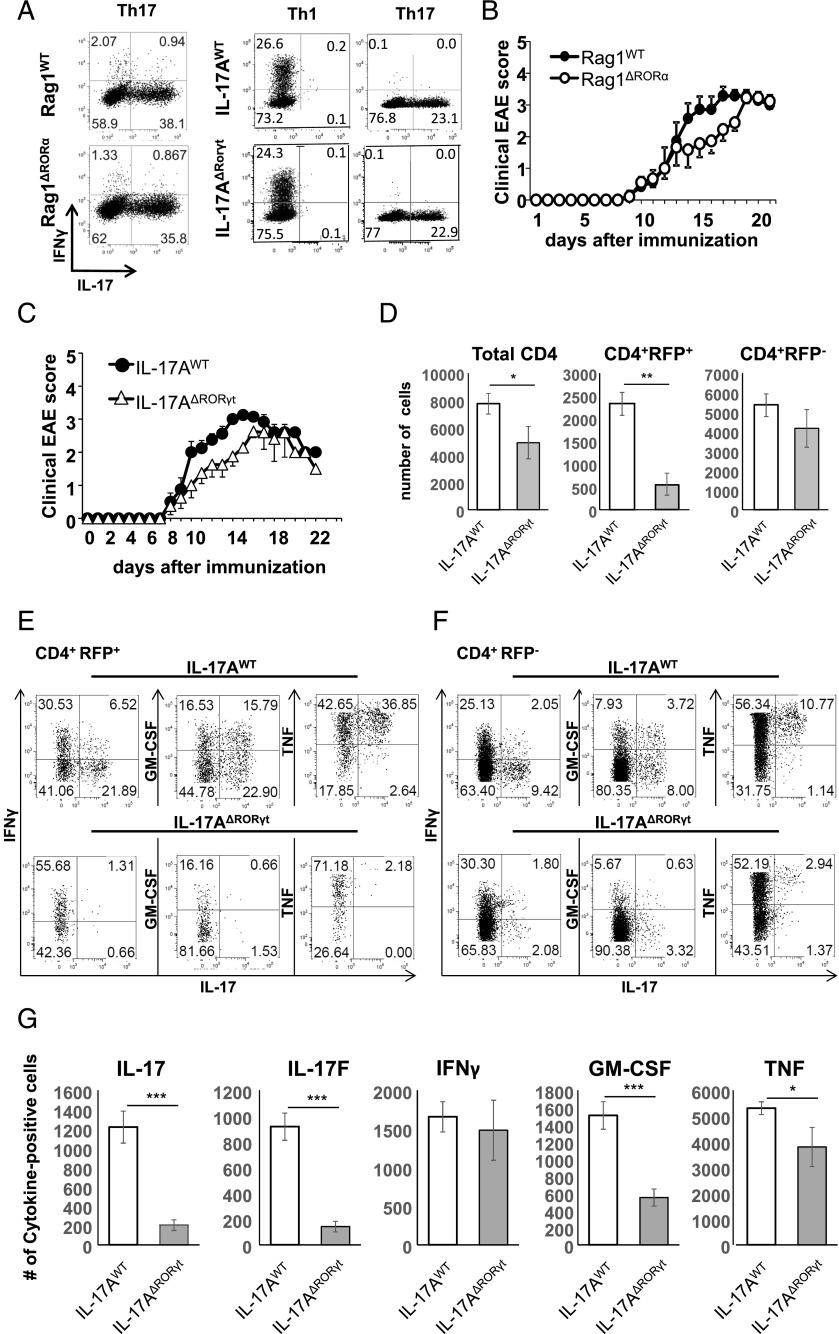
Th17 cell maintenance is not required for EAE immunopathology. (**A**) Flow cytometry for IFN-γ and IL-17 from naive T cells from Rag1^WT^ and Rag1^ΔRORa^ mouse lines polarized in vitro toward Th1 or Th17 cells. (**B**) Clinical EAE scores of Rag1^WT^ and Rag1^ΔRORa^ mouse lines immunized with MOG/CFA (*n* = 6–7). (**C**) Clinical EAE scores of IL-17A^WT^ and IL-17A^ΔRORγt^ mouse lines immunized with MOG/CFA (*n* = 8/group). (**D**) Numbers of total CD4^+^ T cells, RFP^+^ Th17 lineage-positive, and RFP^−^ Th17 lineage-negative cells present in the CNS of IL-17A^WT^ controls (white bars) or IL-17A^ΔRORγt^ mice (gray bars) upon EAE induction at day 17 (averages ± SEM., *n* = 6). (**E** and **F**) Flow cytometry for indicated cytokines on RFP^+^ Th17 lineage-positive (E) or RFP^−^ lineage-negative (F) cells obtained from IL-17A^WT^ and IL-17A^ΔRORγt^ mice undergoing EAE, as shown in (C). (**G**) Numeric presence of total CD4^+^ T cells expressing indicated cytokines in the CNS of IL-17A^WT^ controls (white bars) or IL-17A^ΔRORγt^ (gray bars) during EAE at day 17 (averages ± SEM, *n* = 6). *p < 0.05, **p < 0.01, ***p < 0.001.

Th17 cells rely on the RORα and especially RORγt for their differentiation (9, 49). Hence, the absence of RORγt prevents the differentiation of Th17 cells and susceptibility to EAE (9, 49). However, it is not clear whether these orphan receptors remain important for Th17 cell maintenance. This is of particular importance for potential therapeutic targeting of Th17 cells in inflammatory disorders. Thus, we isolated naive CD4^+^ T cells from Rag1^Cre^ RORα^fl/fl^ Rosa^stop-tdRFP^ (from hereon called Rag1^ΔRORα^), IL-17A^Cre^ RORγt^fl/fl^ Rosa^stop-tdRFP^ (from hereon called IL-17A^ΔRORγt^), and their respective Rag1^WT^ and IL-17A^WT^ controls, and differentiated the cells in vitro toward the Th17 subset. In vitro polarization of naive CD4^+^ T cells from Rag1^ΔRORα^ and IL-17A^ΔRORγt^ cells into Th1 or Th17 was indistinguishable from their respective controls (Fig. 6A). Moreover, IL-17A^Cre^–mediated deletion of *Rorc* did not affect the in vitro proliferation of naive T cells under Th17-polarizing conditions (data not shown).

We next analyzed the susceptibility of Rag1^ΔRORα^ and IL-17A^ΔRORγt^ mice to MOG_35–55_/CFA-induced EAE. The absence of RORα in all lymphocytes in Rag1^ΔRORα^ hosts did not impact EAE onset compared with controls, but the clinical score progression was slightly delayed in the Rag1^ΔRORα^ hosts (Fig. 6B). Similarly, IL-17A^Cre^–mediated deletion of *Rorc* in IL-17A^ΔRORγt^ mice did not significantly delay the onset or final clinical score of EAE, but resulted in a minor delay in disease progression (Fig. 6C). Detailed analysis of the CD4^+^ T cell compartment in the CNS of IL-17A^ΔRORγt^ mice revealed a significant reduction (71% ± 12%) in RFP^+^CD4^+^ T cells compared with that observed in IL-17A^WT^ controls, a number that contributed to the reduction in total numbers of CNS-infiltrating T cells in the former strain (Fig. 6D). The limited number of RFP^+^CD4^+^ T cells remaining in IL-17A^ΔRORγt^ mice did not express IL-17 or IL-17F, but did produce IFN-γ, with reduced proportions of TNF and GM-CSF–positive T cells compared with controls (Fig. 6E). The reduction in GM-CSF–producing cells in IL-17A^ΔRORγt^ mice was not compensated by GM-CSF production from the RFP^−^ T cell CNS infiltrate (Fig. 6F). As a result, the total number of CD4^+^ T cells producing IL-17, IL-17F, GM-CSF, and TNF was significantly reduced in the IL-17A^ΔRORγt^ mice (Fig. 6G). However, no significant difference in numbers of total IFN-γ–producing CNS-infiltrating T cells was found between IL-17A^WT^ and IL-17A^ΔRORγt^ mice (Fig. 6G). These data indicate that Th17 cells require RORγt not only for their initial generation, but also for their IL-17 production and long-term survival. Importantly, once Th17 cells have been generated, the excision of *Rorc* did not significantly affect the clinical outcome of EAE despite the significant reduction in IL-17– and GM-CSF–producing CD4^+^ T cells.

## Discussion

The mechanism underlying the pathogenicity of T cells and the identity of CD4^+^ T cells instrumental for the onset and maintenance of immunopathology, especially those inducing EAE, are still debated in the literature. In this study, we demonstrate that Th17 cells and their Tbet- and IFN-γ–expressing progeny are the predominant populations of T cells present in EAE and *H. hepaticus*-induced typhlocolitis, in line with their established role as potent effector cells contributing to immunity and immunopathology (10, 11). To our knowledge, for the first time, we assessed the influence of the excision of Tbet, Eomes, RORα, and RORγt, in all lymphocytes or in IL-17–expressing cells only, in the development of immunopathology in vivo. We show that neither the IFN-γ–producing Th17 cell progeny (ex-Th17 and IL-17/IFN-γ double producers in the case of EAE, or ex-Th17 cells in the case of *H. hepaticus* colitis) nor long-term Th17 cell maintenance (in the case of EAE) is essential for the establishment of T cell–mediated immunopathology.

Numerous studies have shown that distinct populations of T cell subsets have the capacity to induce pathology upon adoptive transfer into lymphopenic or T cell–sufficient hosts, with different types of EAE as a result (50, 51). However, criticism has been raised that these cells, often bearing an Ag-specific TCR and polarized in vitro with a mix of cytokines, may not accurately recapitulate the phenotype of in vivo generated effector T cells. Our study did not make use of TCR transgenic mice or the transfer of in vitro cultured cells. Instead, we employed conditional deletion, either Rag1^Cre^- or IL-17A^Cre^–mediated, of genes of interest and tracked the Th17 population and its progeny with an RFP lineage marker. We show that the generation of IL-17/IFN-γ double-producing T cells requires the expression of *Tbx21* in Th17 cells during EAE. In stark contrast to the EAE model, we further demonstrate that Tbet is not an absolute requirement for the generation of these double-producing lymphocytes, as these cells were readily found in *H. hepaticus* typhlocolitis in Rag1ΔTbet and IL-17AΔTbet mice. This may highlight the different microenvironments present in the intestine compared with the CNS, providing different cues enabling the development of double-producing T cells. Furthermore, it re-emphasizes the high degree of plasticity of Th17 cells and the extraordinary tailored response of the immune system, depending on microorganisms encountered, as well as the site of inflammation.

IL-17/IFN-γ double-producing T cells have been found during active colitis in mice and humans (18, 21, 52, 53); however, their contribution to intestinal pathology is largely unknown. A recent study by Harbour et al. (24) using adoptive transfer into lymphopenic hosts of in vitro polarized Th17 cells from *Tbx21*^−/−^ mice showed that these cells were unable to induce colitis, despite unaffected in vivo generation of IL-17/IFN-γ double-producing cells in the recipients. In contrast, we have previously demonstrated that IL-17/IFN-γ double-positive T cells isolated from the large intestine of *H. hepaticus*-infected colitic mice are able to induce colitis upon transfer to *H. hepaticus*-infected Rag2^−/−^ mice (16), indicating that ex vivo IL-17/IFN-γ double-producing lymphocytes isolated from Tbet-sufficient mice can induce intestinal pathology. Moreover, as shown in the current study, IL-17A^Cre^– or Rag1^Cre^-mediated excision of *Tbx21* in cells once expressing IL-17 or Rag1 had no effect on the number of IL-17/IFN-γ– double-producing cells, nor on the severity of immunopathology in *H. hepaticus* colitis. Hence, in vivo polarized cells or those encountering specific cues associated with particular pathogens such as *H. hepaticus* may directly contribute to colitis, independently of their ability to express Tbet. Of note, Rag1^Cre^-mediated excision of *Tbx21* did result in the presence of IL-17/IFN-γ double-producing cells in the absence of bona fide Th1 cell development (6), indicating that double producers are most likely Th17 cell derived.

The generation of Th17-derived Th1-like cells, which have lost the expression of IL-17, was dependent on the presence of Tbet in both the *H. hepaticus* typhlocolitis and EAE models. Importantly, the excision of *Tbx21* in IL-17–producing cells had no impact on *H. hepaticus*-induced intestinal pathology, indicating that, in this model, Th17 cell transition to IFN-γ–producing Th1-like cells is not absolutely required for colitis development. These findings are in contrast to those by Harbour et al. (24), who concluded that Tbet expression by Th17 cells is required for their transition to Th1-like cells and for mediating transfer colitis. Among possible explanations for this discrepancy is the use of different colitis models and the use of mice in which *Tbx21* is excised in vivo upon IL-17 or Rag1 expression in our study versus the use of in vitro differentiated Th17 cells from Tbet-deficient mice in the report by Harbour et al. (24). In contrast to the findings in the *H. hepaticus* colitis model, IL-17–mediated deletion of *Tbx21* had a mild reducing impact on EAE. These results are in line with recent studies indicating that pathogenicity of IFN-γ–producing T cells is independent or partially dependent on Tbet (22, 31, 54). Two studies made use of in vitro stimulated and adoptively transferred Ag-specific T cells from Tbx21-deficient mice or CD4^Cre^-mediated *Tbx21* excision (22, 31). Duhen et al. (54) used gene knockout mice or CD4^Cre^-mediated gene excision, affecting several lineages and cell types, including CD8 T cells. We extended these observations by employing *Tbx21* excision specifically in IL-17–producing cells, and conclude that the absence of Tbet in in vivo differentiated Th17 cells has limited impact on immunopathology in the intestine and CNS. In line with CD4^cre^-mediated or germline deletion of *Eomes*, we failed to observe effects of Eomes on Th17 cell polarization, plasticity, or immunopathology in the EAE model (data not shown). The removal of *Tbx21* in all lymphocytes, through Rag1^Cre^-mediated deletion, resulted in a much more pronounced reduction of EAE scores, although clinical symptoms were not completely ameliorated. In this case, both bona fide Th1 cells as well as Th17-derived Th1-like cells were largely absent, yet some immunopathology was still observed. This suggests that even an interplay between Th1- and Th17-derived Th1-like cells is not essential for the development of EAE.

In the current study, to our knowledge, we addressed for the first time whether the maintenance of Th17 cells or their progeny is important for the immunopathology observed in EAE. We found a minor contribution of RORα in EAE, in line with a more essential role of RORγt in Th17 cell differentiation (9, 49). Moreover, the excision of *Rorc* after the generation of Th17 cells resulted in rapid loss of Th17 cells, in line with results from pharmacological inhibition of RORγt (55). This reveals an important role for RORγt in maintaining Th17 cells after their generation in addition to their differentiation. The remaining Th17-derived cells exclusively produced IFN-γ, in line with their loss of *Rorc* that is required for the Th17 lineage program, including the expression of IL-17 and IL-17F (9). Interestingly, despite the significant reduction of cells expressing IL-17, the marked loss of Th17 cell progeny, and cells expressing GM-CSF in the CNS of IL-17A^ΔRORγt^ mice, the onset and pathology of EAE were only mildly affected.

Although no significant reduction in EAE upon IL-17A^Cre^–mediated *Tbx21* or *Rorc* excision was observed, this does not exclude a role for Th17 cells in the initiation of EAE. We found that both MOG Ag specificity and the majority of other cytokines implicated in EAE pathogenicity, such as IFN-γ, TNF, and GM-CSF, were found within the Th17 cell– derived lineages. Upon deletion of Tbet within the Th17 subset, the MOG Ag specificity did remain within the Th17 cell lineage. Although MOG Ag is not the only Ag involved in EAE, it suggests that Th17 cell polarization at the initiation of EAE is sufficient to enable entry to the CNS (56). Moreover, it is clear that factors implicated in immunopathology, such as TNF and GM-CSF, are not exclusive for the Th17 cell–derived lineage found in the CNS. Cytokine profiles were altered within the Th17 cell subsets upon Tbet deletion, modifying the combinations of cytokines secreted by the same T cell. It also remains possible that the absence of Tbet does allow for a partial conversion of Th17 cells, but without terminating IL-17 expression or initiating the Tbet transcriptional program such as IFN-γ expression. Although combinations of cytokines produced by the same cell, such as TNF in combination with either IL-17 or IFN-γ that result in distinct cellular responses (57, 58), were altered upon excision of *Tbx21* or *Rorc*, the effect on immunopathology was limited. Because GM-CSF has been shown to be necessary for the development of EAE (46, 59, 60), the alteration of T cell populations producing GM-CSF/TNF in combination with IL-17 or IFN-γ may not significantly impact on immunopathology.

Extensive studies to find the pathogenicity factor(s) have focused on the Th17 cell subset with potential novel mediators of pathology reported (61). However, inflammation is characterized by diversity in cell subsets, mediators, as well as clinical course and drug responsiveness (51). Important cytokines in the Th1 and Th17 cell axis, with the exception of IL-6 and IL-23, have been reported to be dispensable for the induction and clinical disease progression of EAE (51, 62, 63). Our work implies that a focus on a particular Th subset during the pathology phase of disease may be of limited clinical benefit. In summary, our results contribute to a growing body of evidence that immunopathology cannot be attributed to a single lineage of Th cells. Instead, it is likely that multiple Th cell lineages and immune cell types contribute to immunopathology. Until the identification of a lineage-independent pathogenicity factor, disease-modifying therapies may need to continue to be targeted more broadly.

## References

[1] Mosmann T. R. Cherwinski H. Bond M. W. Giedlin M. A. Coffman R. L. 1986 Two types of murine helper T cell clone. I. Definition according to profiles of lymphokine activities and secreted proteins. J. Immunol. 136: 2348–2357. 2419430

[2] Asano M. Toda M. Sakaguchi N. Sakaguchi S. 1996 Autoimmune disease as a consequence of developmental abnormality of a T cell subpopulation. J. Exp. Med. 184: 387–396. 8760792 10.1084/jem.184.2.387 PMC2192701

[3] Brucklacher-Waldert V. Carr E. J. Linterman M. A. Veldhoen M. 2014 Cellular plasticity of CD4+ T cells in the intestine. Front. Immunol. 5: 488. 25339956 10.3389/fimmu.2014.00488 PMC4188036

[4] Veldhoen M. 2009 The role of T helper subsets in autoimmunity and allergy. Curr. Opin. Immunol. 21: 606–611. 19683910 10.1016/j.coi.2009.07.009

[5] Zygmunt B. Veldhoen M. 2011 T helper cell differentiation more than just cytokines. Adv. Immunol. 109: 159–196. 21569915 10.1016/B978-0-12-387664-5.00005-4

[6] Szabo S. J. Sullivan B. M. Stemmann C. Satoskar A. R. Sleckman B. P. Glimcher L. H. 2002 Distinct effects of T-bet in TH1 lineage commitment and IFN-gamma production in CD4 and CD8 T cells. Science 295: 338–342. 11786644 10.1126/science.1065543

[7] Zheng W. Flavell R. A. 1997 The transcription factor GATA-3 is necessary and sufficient for Th2 cytokine gene expression in CD4 T cells. Cell 89: 587–596. 9160750 10.1016/s0092-8674(00)80240-8

[8] Hori S. Nomura T. Sakaguchi S. 2003 Control of regulatory T cell development by the transcription factor Foxp3. Science 299: 1057–1061. 12522256 10.1126/science.1079490

[9] Ivanov I. I. McKenzie B. S. Zhou L. Tadokoro C. E. Lepelley A. Lafaille J. J. Cua D. J. Littman D. R. 2006 The orphan nuclear receptor RORgammat directs the differentiation program of proinflammatory IL-17+ T helper cells. Cell 126: 1121–1133. 16990136 10.1016/j.cell.2006.07.035

[10] Cua D. J. Sherlock J. Chen Y. Murphy C. A. Joyce B. Seymour B. Lucian L. To W. Kwan S. Churakova T. 2003 Interleukin-23 rather than interleukin-12 is the critical cytokine for autoimmune inflammation of the brain. Nature 421: 744–748. 12610626 10.1038/nature01355

[11] Veldhoen M. Hocking R. J. Flavell R. A. Stockinger B. 2006 Signals mediated by transforming growth factor-beta initiate autoimmune encephalomyelitis, but chronic inflammation is needed to sustain disease. Nat. Immunol. 7: 1151–1156. 16998492 10.1038/ni1391

[12] Acosta-Rodriguez E. V. Rivino L. Geginat J. Jarrossay D. Gattorno M. Lanzavecchia A. Sallusto F. Napolitani G. 2007 Surface phenotype and antigenic specificity of human interleukin 17-producing T helper memory cells. Nat. Immunol. 8: 639–646. 17486092 10.1038/ni1467

[13] Mangan P. R. Harrington L. E. O’Quinn D. B. Helms W. S. Bullard D. C. Elson C. O. Hatton R. D. Wahl S. M. Schoeb T. R. Weaver C. T. 2006 Transforming growth factor-beta induces development of the T(H)17 lineage. Nature 441: 231–234. 16648837 10.1038/nature04754

[14] Cosmi L. De Palma R. Santarlasci V. Maggi L. Capone M. Frosali F. Rodolico G. Querci V. Abbate G. Angeli R. 2008 Human interleukin 17-producing cells originate from a CD161+CD4+ T cell precursor. J. Exp. Med. 205: 1903–1916. 18663128 10.1084/jem.20080397 PMC2525581

[15] Brucklacher-Waldert V. Stuerner K. Kolster M. Wolthausen J. Tolosa E. 2009 Phenotypical and functional characterization of T helper 17 cells in multiple sclerosis. Brain 132: 3329–3341. 19933767 10.1093/brain/awp289

[16] Morrison P. J. Bending D. Fouser L. A. Wright J. F. Stockinger B. Cooke A. Kullberg M. C. 2013 Th17-cell plasticity in *Helicobacter hepaticus*-induced intestinal inflammation. Mucosal Immunol. 6: 1143–1156. 23462910 10.1038/mi.2013.11

[17] Nistala K. Adams S. Cambrook H. Ursu S. Olivito B. de Jager W. Evans J. G. Cimaz R. Bajaj-Elliott M. Wedderburn L. R. 2010 Th17 plasticity in human autoimmune arthritis is driven by the inflammatory environment. Proc. Natl. Acad. Sci. USA 107: 14751–14756. 20679229 10.1073/pnas.1003852107 PMC2930428

[18] Ahern P. P. Schiering C. Buonocore S. McGeachy M. J. Cua D. J. Maloy K. J. Powrie F. 2010 Interleukin-23 drives intestinal inflammation through direct activity on T cells. Immunity 33: 279–288. 20732640 10.1016/j.immuni.2010.08.010 PMC3078329

[19] Bending D. De la Peña H. Veldhoen M. Phillips J. M. Uyttenhove C. Stockinger B. Cooke A. 2009 Highly purified Th17 cells from BDC2.5NOD mice convert into Th1-like cells in NOD/SCID recipient mice. J. Clin. Invest. 119: 565–572. 19188681 10.1172/JCI37865 PMC2648686

[20] Hirota K. Duarte J. H. Veldhoen M. Hornsby E. Li Y. Cua D. J. Ahlfors H. Wilhelm C. Tolaini M. Menzel U. 2011 Fate mapping of IL-17-producing T cells in inflammatory responses. Nat. Immunol. 12: 255–263. 21278737 10.1038/ni.1993 PMC3040235

[21] Lee Y. K. Turner H. Maynard C. L. Oliver J. R. Chen D. Elson C. O. Weaver C. T. 2009 Late developmental plasticity in the T helper 17 lineage. Immunity 30: 92–107. 19119024 10.1016/j.immuni.2008.11.005 PMC3607320

[22] Wang Y. Godec J. Ben-Aissa K. Cui K. Zhao K. Pucsek A. B. Lee Y. K. Weaver C. T. Yagi R. Lazarevic V. 2014 The transcription factors T-bet and Runx are required for the ontogeny of pathogenic interferon-γ-producing T helper 17 cells. Immunity 40: 355–366. 24530058 10.1016/j.immuni.2014.01.002 PMC3965587

[23] Bettelli E. Sullivan B. Szabo S. J. Sobel R. A. Glimcher L. H. Kuchroo V. K. 2004 Loss of T-bet, but not STAT1, prevents the development of experimental autoimmune encephalomyelitis. J. Exp. Med. 200: 79–87. 15238607 10.1084/jem.20031819 PMC2213316

[24] Harbour S. N. Maynard C. L. Zindl C. L. Schoeb T. R. Weaver C. T. 2015 Th17 cells give rise to Th1 cells that are required for the pathogenesis of colitis. Proc. Natl. Acad. Sci. USA 112: 7061–7066. 26038559 10.1073/pnas.1415675112 PMC4460486

[25] Juedes A. E. Rodrigo E. Togher L. Glimcher L. H. von Herrath M. G. 2004 T-bet controls autoaggressive CD8 lymphocyte responses in type 1 diabetes. J. Exp. Med. 199: 1153–1162. 15096540 10.1084/jem.20031873 PMC2211889

[26] Nath N. Prasad R. Giri S. Singh A. K. Singh I. 2006 T-bet is essential for the progression of experimental autoimmune encephalomyelitis. Immunology 118: 384–391. 16827899 10.1111/j.1365-2567.2006.02385.x PMC1782298

[27] Neurath M. F. Weigmann B. Finotto S. Glickman J. Nieuwenhuis E. Iijima H. Mizoguchi A. Mizoguchi E. Mudter J. Galle P. R. 2002 The transcription factor T-bet regulates mucosal T cell activation in experimental colitis and Crohn’s disease. [Published erratum appears in 2002 *J. Exp. Med.* 195: 1513.] J. Exp. Med. 195: 1129–1143. 11994418 10.1084/jem.20011956 PMC2193714

[28] Peng S. L. Szabo S. J. Glimcher L. H. 2002 T-bet regulates IgG class switching and pathogenic autoantibody production. Proc. Natl. Acad. Sci. USA 99: 5545–5550. 11960012 10.1073/pnas.082114899 PMC122806

[29] Wang J. Fathman J. W. Lugo-Villarino G. Scimone L. von Andrian U. Dorfman D. M. Glimcher L. H. 2006 Transcription factor T-bet regulates inflammatory arthritis through its function in dendritic cells. J. Clin. Invest. 116: 414–421. 16410834 10.1172/JCI26631 PMC1326147

[30] Grifka-Walk H. M. Lalor S. J. Segal B. M. 2013 Highly polarized Th17 cells induce EAE via a T-bet independent mechanism. Eur. J. Immunol. 43: 2824–2831. 23878008 10.1002/eji.201343723 PMC3838449

[31] O’Connor R. A. Cambrook H. Huettner K. Anderton S. M. 2013 T-bet is essential for Th1-mediated, but not Th17-mediated, CNS autoimmune disease. Eur. J. Immunol. 43: 2818–2823. 23878019 10.1002/eji.201343689 PMC4068221

[32] McCormack M. P. Forster A. Drynan L. Pannell R. Rabbitts T. H. 2003 The LMO2 T-cell oncogene is activated via chromosomal translocations or retroviral insertion during gene therapy but has no mandatory role in normal T-cell development. Mol. Cell. Biol. 23: 9003–9013. 14645513 10.1128/MCB.23.24.9003-9013.2003 PMC309712

[33] Hao Z. Rajewsky K. 2001 Homeostasis of peripheral B cells in the absence of B cell influx from the bone marrow. J. Exp. Med. 194: 1151–1164. 11602643 10.1084/jem.194.8.1151 PMC2193512

[34] Stetson D. B. Mohrs M. Reinhardt R. L. Baron J. L. Wang Z. E. Gapin L. Kronenberg M. Locksley R. M. 2003 Constitutive cytokine mRNAs mark natural killer (NK) and NK T cells poised for rapid effector function. J. Exp. Med. 198: 1069–1076. 14530376 10.1084/jem.20030630 PMC2194220

[35] Intlekofer A. M. Banerjee A. Takemoto N. Gordon S. M. Dejong C. S. Shin H. Hunter C. A. Wherry E. J. Lindsten T. Reiner S. L. 2008 Anomalous type 17 response to viral infection by CD8+ T cells lacking T-bet and eomesodermin. Science 321: 408–411. 18635804 10.1126/science.1159806 PMC2807624

[36] Oliphant C. J. Hwang Y. Y. Walker J. A. Salimi M. Wong S. H. Brewer J. M. Englezakis A. Barlow J. L. Hams E. Scanlon S. T. 2014 MHCII-mediated dialog between group 2 innate lymphoid cells and CD4(+) T cells potentiates type 2 immunity and promotes parasitic helminth expulsion. Immunity 41: 283–295. 25088770 10.1016/j.immuni.2014.06.016 PMC4148706

[37] Veldhoen M. Hocking R. J. Atkins C. J. Locksley R. M. Stockinger B. 2006 TGFbeta in the context of an inflammatory cytokine milieu supports de novo differentiation of IL-17-producing T cells. Immunity 24: 179–189. 16473830 10.1016/j.immuni.2006.01.001

[38] Ward J. M. Anver M. R. Haines D. C. Benveniste R. E. 1994 Chronic active hepatitis in mice caused by *Helicobacter hepaticus*. Am. J. Pathol. 145: 959–968. 7943185 PMC1887338

[39] Fox J. G. Dewhirst F. E. Tully J. G. Paster B. J. Yan L. Taylor N. S. Collins M. J. Jr. Gorelick P. L. Ward J. M. 1994 *Helicobacter hepaticus* sp. nov., a microaerophilic bacterium isolated from livers and intestinal mucosal scrapings from mice. J. Clin. Microbiol. 32: 1238–1245. 8051250 10.1128/jcm.32.5.1238-1245.1994 PMC263656

[40] Kullberg M. C. Jankovic D. Feng C. G. Hue S. Gorelick P. L. McKenzie B. S. Cua D. J. Powrie F. Cheever A. W. Maloy K. J. Sher A. 2006 IL-23 plays a key role in *Helicobacter hepaticus*-induced T cell-dependent colitis. J. Exp. Med. 203: 2485–2494. 17030948 10.1084/jem.20061082 PMC2118119

[41] Reinhardt R. L. Liang H. E. Locksley R. M. 2009 Cytokine-secreting follicular T cells shape the antibody repertoire. Nat. Immunol. 10: 385–393. 19252490 10.1038/ni.1715 PMC2714053

[42] Becher B. Segal B. M. 2011 T(H)17 cytokines in autoimmune neuro-inflammation. Curr. Opin. Immunol. 23: 707–712. 21907555 10.1016/j.coi.2011.08.005 PMC3535446

[43] Peters A. Lee Y. Kuchroo V. K. 2011 The many faces of Th17 cells. Curr. Opin. Immunol. 23: 702–706. 21899997 10.1016/j.coi.2011.08.007 PMC3232281

[44] Pearce E. L. Mullen A. C. Martins G. A. Krawczyk C. M. Hutchins A. S. Zediak V. P. Banica M. DiCioccio C. B. Gross D. A. Mao C. A. 2003 Control of effector CD8+ T cell function by the transcription factor Eomesodermin. Science 302: 1041–1043. 14605368 10.1126/science.1090148

[45] Hirota K. Turner J. E. Villa M. Duarte J. H. Demengeot J. Steinmetz O. M. Stockinger B. 2013 Plasticity of Th17 cells in Peyer’s patches is responsible for the induction of T cell-dependent IgA responses. Nat. Immunol. 14: 372–379. 23475182 10.1038/ni.2552 PMC3672955

[46] Ponomarev E. D. Shriver L. P. Maresz K. Pedras-Vasconcelos J. Verthelyi D. Dittel B. N. 2007 GM-CSF production by autoreactive T cells is required for the activation of microglial cells and the onset of experimental autoimmune encephalomyelitis. J. Immunol. 178: 39–48. 17182538 10.4049/jimmunol.178.1.39

[47] Kebir H. Ifergan I. Alvarez J. I. Bernard M. Poirier J. Arbour N. Duquette P. Prat A. 2009 Preferential recruitment of interferon-gamma-expressing TH17 cells in multiple sclerosis. Ann. Neurol. 66: 390–402. 19810097 10.1002/ana.21748

[48] Yang Y. Weiner J. Liu Y. Smith A. J. Huss D. J. Winger R. Peng H. Cravens P. D. Racke M. K. Lovett-Racke A. E. 2009 T-bet is essential for encephalitogenicity of both Th1 and Th17 cells. J. Exp. Med. 206: 1549–1564. 19546248 10.1084/jem.20082584 PMC2715092

[49] Yang X. O. Pappu B. P. Nurieva R. Akimzhanov A. Kang H. S. Chung Y. Ma L. Shah B. Panopoulos A. D. Schluns K. S. 2008 T helper 17 lineage differentiation is programmed by orphan nuclear receptors ROR alpha and ROR gamma. Immunity 28: 29–39. 18164222 10.1016/j.immuni.2007.11.016 PMC2587175

[50] Jäger A. Dardalhon V. Sobel R. A. Bettelli E. Kuchroo V. K. 2009 Th1, Th17, and Th9 effector cells induce experimental autoimmune encephalomyelitis with different pathological phenotypes. J. Immunol. 183: 7169–7177. 19890056 10.4049/jimmunol.0901906 PMC2921715

[51] Kroenke M. A. Carlson T. J. Andjelkovic A. V. Segal B. M. 2008 IL-12- and IL-23-modulated T cells induce distinct types of EAE based on histology, CNS chemokine profile, and response to cytokine inhibition. J. Exp. Med. 205: 1535–1541. 18573909 10.1084/jem.20080159 PMC2442630

[52] Annunziato F. Cosmi L. Santarlasci V. Maggi L. Liotta F. Mazzinghi B. Parente E. Filì L. Ferri S. Frosali F. 2007 Phenotypic and functional features of human Th17 cells. J. Exp. Med. 204: 1849–1861. 17635957 10.1084/jem.20070663 PMC2118657

[53] Huber S. Gagliani N. Esplugues E. O’Connor W. Jr. Huber F. J. Chaudhry A. Kamanaka M. Kobayashi Y. Booth C. J. Rudensky A. Y. 2011 Th17 cells express interleukin-10 receptor and are controlled by Foxp3⁻ and Foxp3+ regulatory CD4+ T cells in an interleukin-10-dependent manner. Immunity 34: 554–565. 21511184 10.1016/j.immuni.2011.01.020 PMC3113617

[54] Duhen R. Glatigny S. Arbelaez C. A. Blair T. C. Oukka M. Bettelli E. 2013 Cutting edge: the pathogenicity of IFN-γ-producing Th17 cells is independent of T-bet. J. Immunol. 190: 4478–4482. 23543757 10.4049/jimmunol.1203172 PMC3633668

[55] Withers D. R. Hepworth M. R. Wang X. Mackley E. C. Halford E. E. Dutton E. E. Marriott C. L. Brucklacher-Waldert V. Veldhoen M. Kelsen J. 2016 Transient inhibition of ROR-γt therapeutically limits intestinal inflammation by reducing TH17 cells and preserving group 3 innate lymphoid cells. Nat. Med. 22: 319–323. 26878233 10.1038/nm.4046 PMC4948756

[56] Reboldi A. Coisne C. Baumjohann D. Benvenuto F. Bottinelli D. Lira S. Uccelli A. Lanzavecchia A. Engelhardt B. Sallusto F. 2009 C-C chemokine receptor 6-regulated entry of TH-17 cells into the CNS through the choroid plexus is required for the initiation of EAE. Nat. Immunol. 10: 514–523. 19305396 10.1038/ni.1716

[57] Griffin G. K. Newton G. Tarrio M. L. Bu D. X. Maganto-Garcia E. Azcutia V. Alcaide P. Grabie N. Luscinskas F. W. Croce K. J. Lichtman A. H. 2012 IL-17 and TNF-α sustain neutrophil recruitment during inflammation through synergistic effects on endothelial activation. J. Immunol. 188: 6287–6299. 22566565 10.4049/jimmunol.1200385 PMC3370121

[58] Liu Y. Wang L. Kikuiri T. Akiyama K. Chen C. Xu X. Yang R. Chen W. Wang S. Shi S. 2011 Mesenchymal stem cell-based tissue regeneration is governed by recipient T lymphocytes via IFN-γ and TNF-α. Nat. Med. 17: 1594–1601. 22101767 10.1038/nm.2542 PMC3233650

[59] Codarri L. Gyülvészi G. Tosevski V. Hesske L. Fontana A. Magnenat L. Suter T. Becher B. 2011 RORγt drives production of the cytokine GM-CSF in helper T cells, which is essential for the effector phase of autoimmune neuroinflammation. Nat. Immunol. 12: 560–567. 21516112 10.1038/ni.2027

[60] El-Behi M. Ciric B. Dai H. Yan Y. Cullimore M. Safavi F. Zhang G. X. Dittel B. N. Rostami A. 2011 The encephalitogenicity of T(H)17 cells is dependent on IL-1- and IL-23-induced production of the cytokine GM-CSF. Nat. Immunol. 12: 568–575. 21516111 10.1038/ni.2031 PMC3116521

[61] Gaublomme J. T. Yosef N. Lee Y. Gertner R. S. Yang L. V. Wu C. Pandolfi P. P. Mak T. Satija R. Shalek A. K. 2015 Single-cell genomics unveils critical regulators of Th17 cell pathogenicity. Cell 163: 1400–1412. 26607794 10.1016/j.cell.2015.11.009 PMC4671824

[62] Ferber I. A. Brocke S. Taylor-Edwards C. Ridgway W. Dinisco C. Steinman L. Dalton D. Fathman C. G. 1996 Mice with a disrupted IFN-gamma gene are susceptible to the induction of experimental autoimmune encephalomyelitis (EAE). J. Immunol. 156: 5–7. 8598493

[63] Haak S. Croxford A. L. Kreymborg K. Heppner F. L. Pouly S. Becher B. Waisman A. 2009 IL-17A and IL-17F do not contribute vitally to autoimmune neuro-inflammation in mice. J. Clin. Invest. 119: 61–69. 19075395 10.1172/JCI35997 PMC2613466

